# Structure and function of dioxygenases in histone demethylation and DNA/RNA demethylation

**DOI:** 10.1107/S2052252514020922

**Published:** 2014-10-28

**Authors:** Cheng Dong, Heng Zhang, Chao Xu, Cheryl H. Arrowsmith, Jinrong Min

**Affiliations:** a Structural Genomics Consortium, University of Toronto, 101 College Street, Toronto, Ontario M5G 1L7, Canada; b Ontario Cancer Institute and Department of Medical Biophysics, University of Toronto, Toronto, Ontario M5G 2M9, Canada; c Department of Physiology, University of Toronto, Toronto, Ontario M5S 1A8, Canada

**Keywords:** dioxygenases, histone demethyl­ation, DNA/RNA demethylation, *N*^6^-methyl­adenosine, ALKBH5

## Abstract

Iron(II) and 2-oxoglutarate (2OG)-dependent dioxygenases involved in histone and DNA/RNA demethylation convert the cosubstrate 2OG and oxygen to succinate and carbon dioxide, resulting in hydroxylation of the methyl group of the substrates and subsequent demethylation. Recent evidence has shown that these 2OG dioxygenases play vital roles in a variety of biological processes, including transcriptional regulation and gene expression. In this review, the structure and function of these dioxygenases in histone and nucleic acid demethylation will be discussed. Given the important roles of these 2OG dioxygenases, detailed analysis and comparison of the 2OG dioxygenases will guide the design of target-specific small-molecule chemical probes and inhibitors.

## Abstract 2

The structure and function of dioxygenases in histone demethylation and DNA/RNA dimethylation are discussed.

## 1. Introduction   

Histone methylation together with DNA and RNA methyl­ation are the centerpieces in epigenetic inheritance, and play pivotal roles in the epigenetic regulation of transcriptional activation and repression (Shi, 2007 ▶; Fu & He, 2012 ▶). Histone lysine (K) methylation often occurs on H3K4, K9, K27, K36, K79 and H4K20, which can be mono-, di- and trimethylated at the ∊-amino group by the SET domain histone methyltransferase with just one exception Dot1L, which harbors a canonical open α/β catalytic domain, like that of the catalytic domain of protein arginine methyltransferases and DNA methyltransferases (Martin & Zhang, 2005 ▶; Min *et al.*, 2003 ▶). While DNA methylation usually occurs at the 5-carbon position of the cytosine ring (5mC) in a CpG dinucleotide context (Bird, 2002 ▶), the epigenetic RNA methylation typically marks the adenosine N6 position (m^6^A) of mRNA and long noncoding RNA (Grosjean, 2005 ▶; Wei *et al.*, 1975 ▶).

Recent studies have indicated that histone lysine methyl­ation as well as DNA/RNA methylation is dynamically modified in order to regulate the methylation and demethyl­ation levels in cells (Jia *et al.*, 2011 ▶; Zheng *et al.*, 2013 ▶; Song & He, 2012 ▶; Mosammaparast & Shi, 2010 ▶; Shi & Tsukada, 2013 ▶). It is worth noting that some Fe^II^ and 2-oxoglutarate (2OG) dioxygenases serve as demethylases of histone and DNA/RNA. Fe^II^- and 2OG-dependent dioxygenases require ferrous iron as a cofactor and 2OG, namely α-ketoglutarate (αKG), as a cosubstrate for their catalytic activity (Hausinger, 2004 ▶). These dioxygenases contain a conserved double-stranded β-helix (DSBH or jelly-roll) domain, in which the Fe^II^ and 2OG are coordinated by some invariant residues (Clifton *et al.*, 2006 ▶). Moreover, the observed distance between an oxidized substrate atom and iron is approximately 4.5 Å in most dioxygenases (Aik *et al.*, 2012 ▶).

Up to now, a growing number of dioxygenases have been identified that can act on histones or DNA/RNA as an oxidized substrate. Structural studies revealed that these Fe^II^- and 2OG-dependent dioxygenases not only contain a conserved DSBH domain, but also have some auxiliary functional domains (Klose *et al.*, 2006 ▶; Tsukada, 2012 ▶).

This review focuses on the structure and function of the three kinds of epigenetic demethylases based on different substrates namely methylated histone lysine, DNA and RNA. In addition to dioxygenase-based JmjC-domain-containing histone demethylases, a pair of amine oxidases (LSD1 and LSD2) can also demethylate histone lysine H3K4, and these two classes of histone demethylases have different catalytic mechanisms. Interestingly, a non-dioxygenase protein LOXL2 was also reported to be able to remove the methylation of H3K4 by deaminating the target lysine (Herranz *et al.*, 2012 ▶). Ten-eleven translocation proteins TET1–3 act as 5-methyl­cytosine hydroxylases and were found to promote active DNA demethylation (Xu *et al.*, 2012 ▶) and, so far, only two members, FTO and ALKBH5, have been reported to be involved in m^6^A RNA demethylation (Zheng *et al.*, 2013 ▶; Jia *et al.*, 2011 ▶).

## 2. Histone demethylation   

Histone lysine methylation plays a critical role in regulating various biological processes, such as transcriptional regulation, DNA replication and DNA repair (Martin & Zhang, 2005 ▶; Zhang & Reinberg, 2001 ▶). The methylation status of the lysine includes mono-, di- and trimethylation. Histone methylation had been considered to be irreversible for a long time until the first histone demethylase, lysine-specific demethylase I (LSD1) was identified in 2004 by Shi *et al.* (Shi *et al.*, 2004 ▶). Subsequently, a Jumonji-like domain C-terminus (JmjC) domain-containing demethylase was identified by Tsukada *et al.* (2006 ▶). Therefore, histone lysine methylation is a dynamic and reversible modification through the balancing of histone lysine methyltransferases and histone lysine demethylases (KDMs) (Shi & Whetstine, 2007 ▶).

## 3. LSD family   

LSD1 is a flavin adenine dinucleotide (FAD)-dependent amine oxidase. In this catalytic reaction, LSD1 utilizes FAD as a cofactor to catalyze the amine oxidation of the methylated lysine, creating an imine intermediate. The imine intermediate is spontaneously hydrolyzed to form an unstable carbinol­amine intermediate, which releases formaldehyde and generates unmethylated lysine (Shi *et al.*, 2004 ▶). However, lysine (K) residues exit in three methylated states, namely mono-, di- or trimethylated lysine (Kme1, Kme2 and Kme3). This reaction mechanism requires protonated nitrogen on the ∊-amino group of methylated lysine in order to form the imine intermediate, but the nitrogen of trimethylated lysine cannot be protonated. Therefore, LSD1 can demethylate Kme1 and Kme2 but not Kme3.

**Figure 1:**
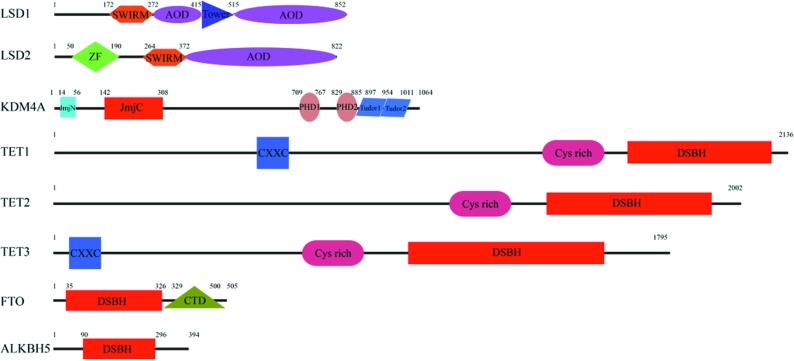
Functional domain structures of human demethylases: human LSD1/KDM1A (UniProtKB: O60341), human LSD2/KDM1B (Q8NB78), human KDM4A/JHDM3A/JMJD2A (O75164), human TET1 (Q8NFU7), human TET2 (Q6N021), human TET3 (K9JJH7), FTO (Q9C0B1) and ALKBH5 (Q6P6C2). SWIRM is Swi3p, Rsc8p and Moira domain, AOD is amine oxidase domain, Tower is tower domain, ZF is zinc-finger domain, JmjN is Jumonji N domain, JmjC is Jumonji C domain, PHD is plant homeodomain, Tudor is Tudor domain, CXXC is CXXC zinc-finger domain, Cys-rich is cysteine-rich domain, DSBH is double-stranded β-helix and CTD is C-terminal domain.

The LSD family includes two members: LSD1 (also known as KDM1A) and LSD2 (also known as KDM1B), and their crystal structures in complex with substrates have been determined and the demethylation mechanism has also been studied (Yang *et al.*, 2006 ▶; Forneris *et al.*, 2007 ▶; Zhang *et al.*, 2013 ▶; Fang *et al.*, 2013 ▶). Crystallographic studies show that LSD1 and LSD2 share a conserved C-terminal amine oxidase domain (AOD) and a SWIRM (Swi3p, Rsc8p and Moira) domain. In addition, LSD1 has a unique insertion (referred to as the Tower domain) that is embedded in the AOD domain (Forneris *et al.*, 2007 ▶). On the other hand, LSD2 has a unique zinc-finger domain at the N-terminus (Figs. 1 ▶, 2 ▶ *a* and 2 ▶ *b*) (Zhang *et al.*, 2013 ▶). The conserved AOD domain contains two lobes: one is used for FAD binding, and the other is responsible for substrate binding. The interface of the two lobes forms a large highly negatively charged catalytic cavity, which is large enough to accommodate a large segment of H3 tail, suggesting that the inability of LSD1 and LSD2 to demethyl­ate trimethylated lysine is not owing to structural steric hindrance, but rather because of the inherent limitations of its chemical mechanism. The SWIRM domain adopts an α-helical fold that packs against the FAD-binding lobe of AOD domain. The SWIRM domain is responsible for maintaining the integrity of the overall core structure and is required for enzymatic activity (Yang *et al.*, 2006 ▶; Zhang *et al.*, 2013 ▶). The Tower domain of LSD1 is required for binding to CoREST (co-repressor of RE1-silencing transcription factor), which enables LSD1 to demethylate nucleosomes through the interactions between CoREST and nucleosomal DNA (Yang *et al.*, 2006 ▶). Unlike LSD1, LSD2 is able to independently work on demethylation of nucleosomal substrates and the zinc-finger domain is required for LSD2 demethylase activity (Zhang *et al.*, 2013 ▶; Fang *et al.*, 2010 ▶; Yang, Jiang *et al.*, 2010 ▶).

## 4. JmjC family   

The JmjC family contains several subfamilies, which belong to the 2OG and ferrous iron-dependent dioxygenases (Cloos *et al.*, 2008 ▶; Nottke *et al.*, 2009 ▶). The JmjC domain family of demethylases uses 2OG and Fe^II^ as cofactors in the presence of molecular oxygen to hydroxylate the methylated histone, producing succinate, CO_2_ and unstable carbinolamine; subsequently carbinolamine spontaneously releases formaldehyde and generates demethylated lysine. Unlike LSD-mediated demethyl­ation, the JmjC family is also able to demethylate trimethylated lysine (Tsukada *et al.*, 2006 ▶; Klose *et al.*, 2006 ▶).

Histone lysine methylation has specific physiological functions depending on the lysine modification sites and the degree of methylation (Martin & Zhang, 2005 ▶; Mosammaparast & Shi, 2010 ▶). JmjC domain-containing proteins exhibit different substrate preferences *via* their specific catalytic site architectures. Recent studies show that there are more than 30 different proteins that contain a JmjC domain in the human genome, and most of them have been proved to function as histone demethylases. The modification of H3K79 is unique and its specific demethylases have not yet been discovered (Shi & Tsukada, 2013 ▶).

Structural studies revealed that the conserved JmjC domain consists of eight β-strands, which form a jelly-roll fold (also known as double-stranded β-helix or DSBH) and belong to the cupin superfamily of metalloenzymes (Aik *et al.*, 2012 ▶; Ng *et al.*, 2007 ▶) (Fig. 3 ▶ *a*). The active center is buried in the interior of the JmjC domain, where Fe^2+^ is coordinated by three conserved residues (H…D/E…H motif) and further stabilized by the 2OG cofactor (Fig. 3 ▶ *b*). The substrate is bound by unusual C—H⋯O hydrogen bonds, which is formed between one methyl group of methylated lysine and the oxygen atoms from active-site residues (Couture *et al.*, 2007 ▶) (Fig. 3 ▶ *c*).

In addition to the core structure of the JmjC domain, most JmjC-dependent demethylases contain auxiliary functional domains that contribute to substrate recognition, recruitment and catalysis (Fig. 1 ▶, *e.g.* KDM4A), in addition to maintaining the overall structural stability. These auxiliary domains include JmjN, PHD, Tudor, CXXC, bright/arid, FBOX, zinc finger, TPR *etc*. The JmjN domain, which is formed by three short helices and two β-strands, is essential for the demethylation activity of JHDM3/JMJD2 (Chen *et al.*, 2006 ▶). In *C. elegans* KDM7A, the PHD domain (plant homeo domain) is required for demethylase activity towards both H3K9me2 and H3K27me2 by specifically binding to H3K4me3 (Lin *et al.*, 2010 ▶). The PHD domain of human PHF8 and KDM7A exhibits different orientations relative to the catalytic JmjC domain when interacting with a single peptide containing both H3K4me3 and H3K9me2. In other words, PHF8 adopts a bent conformation, allowing the PHD domain to interact with H3K4me3 while the catalytic JmjC domain acts on the H3K9me2 substrate (Qi *et al.*, 2010 ▶). On the contrary, KDM7A adopts an extended conformation, therefore the JmjC domain cannot reach H3K9me2 when the PHD domain binds to H3K4me3 (Yang, Hu *et al.*, 2010 ▶; Horton *et al.*, 2010 ▶; Hou & Yu, 2010 ▶). The CXXC and bright/arid domains are responsible for DNA binding (Patsialou *et al.*, 2005 ▶; Wu *et al.*, 2013 ▶).

## 5. Inhibitors   

**Figure 2:**
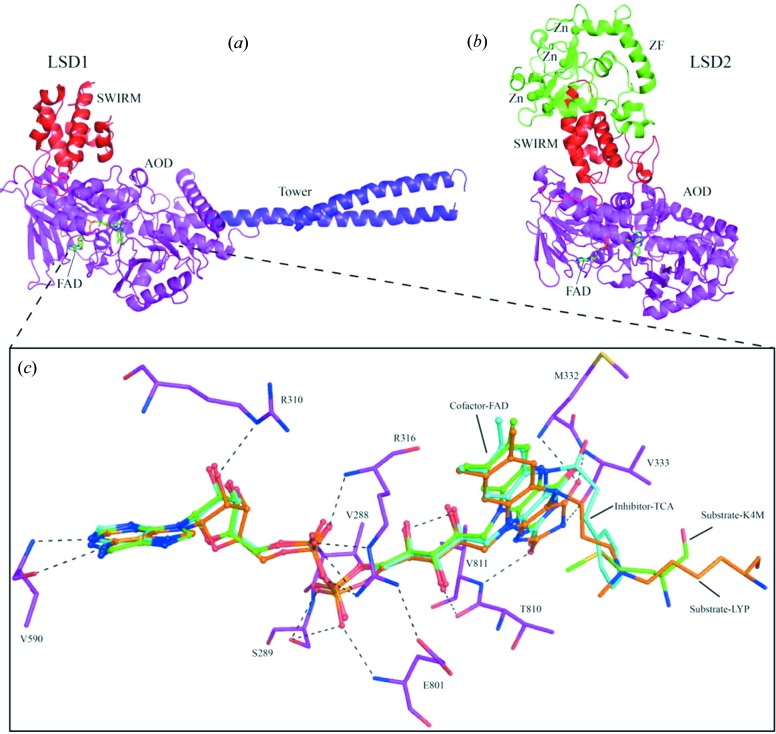
Structural characterization of LSD family. Overall structure of human (*a*) LSD1 (PDB code: 2h94) and (*b*) LSD2 (PDB code: 4gu1) in complex with cofactor FAD, respectively. FAD is shown in sticks with carbon in green. Three zinc atoms are shown as green spheres. The structural domains are indicated and the color scheme corresponds to that used in Fig. 1 ▶. (*c*) Close-up view of the coordination of the cofactor FAD (PDB code: 2uxx), mimetic substrate (PDB codes: 2v1d and 2uxn) and FAD adduct of inhibitor (PDB code: 2xaj) in the active sites of LSD1. FAD is shown as ball-and-stick and the hydrogen bonds contributed by FAD and LSD1 residues are displayed as dashed lines. Substrate-K4M: the catalytic substrate K4 of H3K4me1/2 mutated to methionine. Substrate-LYP: the catalytic substrate K4 analog (*N* ^6^-methyl-*N* ^6^-propyl-l-lysine). Inhibitor-TCA interacts with FAD, forming a covalent adduct (FAD–tranylcypromine adduct). FAD–TCA adduct is shown in sticks with carbon in cyan.

Overexpression of histone demethylases is associated with multiple kinds of human disease, especially tumorigenesis (Højfeldt *et al.*, 2013 ▶). Therefore, inhibitors of histone de­methylases have recently been actively developed. These inhibitors are often designed to mimic the preferred cofactor/substrate. The tranylcypromine (PCPA) is reported to inhibit LSD1 catalysis *via* forming acovalent adducts with cosubstrate FAD in the active site (Schmidt & McCafferty, 2007 ▶; Yang *et al.*, 2007 ▶) (Fig. 2 ▶ *c*). Lots of inhibitors have been identified based on tranylcypromine analogs, such as phenelzine and pargyline. In addition, a series of mimic peptides and polyamine-based inhibitors have also been improved to inhibit LSD1 (Thinnes *et al.*, 2014 ▶).

**Figure 3:**
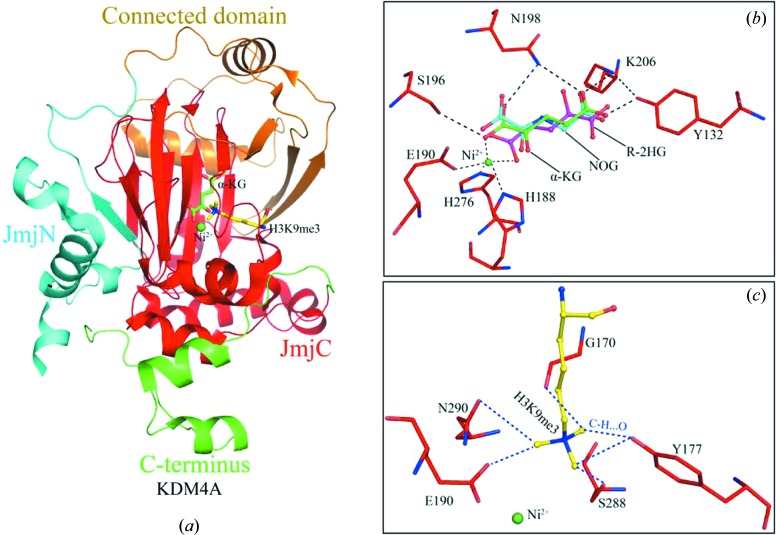
Structural characterization of KDM4A in JmjC family. (*a*) Cartoon representation of FTO in complex with cofactor Ni^2+^, cosubstrate αKG and catalytic substrate H3K9me3 (PDB code: 2q8c). Ni^2+^ is shown as green sphere. αKG and H3K9me3 are shown in sticks with carbon in green and yellow, respectively. The structural domains are indicated in similar color. Connected domain: the region that connecting JmjN domain to JmjC domain. (*b*) Close-up view of the coordination of the cosubstrate αKG (PDB code: 2q8c) and 2OG-based inhibitors NOG/*R*-2HG (PDB codes: 2oq6 and 2ybk) in the active sites of KDM4A. The hydrogen bonds between KDM4A residues with Ni^2+^ or αKG are displayed as dashed lines. (*c*) Close-up view of the coordination of the catalytic substrate H3K9me3. The C—H⋯O hydrogen bonds, formed by one methyl group of methylated lysine and the oxygen atoms from active-site residues are displayed as dashed blue lines.

*N*-Oxalylglycine (NOG) (an analog of 2OG) is a common inhibitor to block the activity of JmjC family *via* hindering oxygen binding to iron. These 2OG derivatives (*e.g.* NOG, *S*/*R*-2HG) have been used to capture inactive states of these enzymes in crystallographic studies. Structural studies show that NOG is coordinated by chelating a divalent metal ion and further stabilized by some residues (Fig. 3 ▶ *b*). Some metal ion chelator-based inhibitors have been reported as competitive inhibitors of 2OG, such as 8-hydroxyquinolines, pyri­dine­dicarboxylic acid (PDCA), daminozide and GSK-J1 (Højfeldt *et al.*, 2013 ▶; Thinnes *et al.*, 2014 ▶).

## 6. DNA demethylation   

DNA methylation often occurs at the 5-carbon position of cytosine (5mC) in a CpG dinucleotide context, which is a key epigenetic mark crucial for genomic regulation and developmental processes in mammals (Bird, 2002 ▶; Suzuki & Bird, 2008 ▶). DNA methylation is a dynamic process controlled by DNA methyltransferases and demethylases (Song & He, 2012 ▶; Hahn *et al.*, 2013 ▶; Smith & Meissner, 2013 ▶). In 1953, 5-hydroxymethylcytosine (5hmc) was first identified in T-even bacteriophages (Wyatt & Cohen, 1953 ▶), but it was not until 2009 that the ten-eleven translocation (TET) proteins were demonstrated to be the enzymes (dioxygenases) that could catalyze the oxidation of 5mC to 5hmc (Tahiliani *et al.*, 2009 ▶). Further research has shown that the TET family of proteins not only convert 5mC to 5hmc, but also can further oxidize 5hmc into 5-formyl­cytosine (5fC) and 5-carboxylcytosine (5cac) in genomic DNA (He *et al.*, 2011 ▶; Ito *et al.*, 2010 ▶; Pfaffeneder *et al.*, 2014 ▶). However, the unmethylated cytosine (C) is not regenerated through the oxidative reactions performed by TET proteins. In other words, the process of 5mC in DNA demethylation cannot be completed by TET proteins themselves. Recent studies suggest that both active and passive mechanisms (Inoue & Zhang, 2011 ▶; Inoue *et al.*, 2011 ▶) are involved in the regulation of DNA demethylation. In the active DNA de­methylation pathways, 5cac can be recognized and excized by thymine DNA glycosylase (TDG) (He *et al.*, 2011 ▶). Alternatively, 5hmc can be converted to 5-hydroxymethyluracil (5hmu) by activation-induce deaminase and further converted to unmodified cytosine by TDG or SMUG1 (single-strand selective monofunctional uracil-DNA glycosylase 1) through the base excision repair pathway (Guo *et al.*, 2011 ▶; Cortellino *et al.*, 2011 ▶; Gavin *et al.*, 2013 ▶). Thus, TET-oxidized 5hmc is a prerequisite step for the active DNA demethylation pathway.

## 7. Potential catalytic mechanism   

The TET family proteins belong to 2-oxoglutarate (2OG) and ferrous iron-dependent dioxygenases. Their catalytic reaction requires 2OG as a cosubstrate and Fe^II^ as a cofactor. In the oxidation reaction, TET protein coordinates one oxygen atom from molecular oxygen (O_2_) into the substrate, yielding hydroxylation of the substrate, and the other oxygen atom into 2OG, leading to a decarboxylation of 2OG and a release of succinate and carbon dioxide (CO_2_) (Xiao *et al.*, 2012 ▶).

## 8. Sequence analysis of TET family proteins   

The members of the mammalian TET family include TET1, TET2 and TET3, which are located on chromosomes 10q21, 4q24 and 2p13 coding for 2136, 2002 and 1795/1660 amino acids, respectively (Mohr *et al.*, 2011 ▶). The TET proteins share a conserved C-terminal catalytic domain. The catalytic domain or CD domain contains Cys-rich and DSBH regions that are conserved in the cupin-like dioxygenase superfamily (Fig. 1 ▶). However, the CD domain includes an unknown spacer region (a large low-complexity insert), which is supposed to be a variable and unstructured region (Tahiliani *et al.*, 2009 ▶). In addition, there is a conserved CXXC zinc-finger domain used for DNA-binding in TET1 and TET3, but not in TET2 (Fig. 1 ▶). The CXXC domain of TET1 and TET3 both lack a ‘KFGG’ motif compared with that of the other CXXC domain family members (Allen *et al.*, 2006 ▶; Xu *et al.*, 2011 ▶).

## 9. CXXC domain   

The human TET CXXC domain is highly conserved and important for recognizing the CpG dinucleotides. The TET1 CXXC domain can bind to unmodified C as well as 5mC and 5hmC-modified CpG-rich DNA, but does not exhibit binding to CG-deleted oligonucleotides (Xu *et al.*, 2011 ▶). However, an unmodified cytosine is absolutely required for the TET3 CXXC domain to interact with DNA and the CXXC domain strongly binds to cytosine-containing DNA with a slight preference for CpG-rich DNA, but does not bind to the A/T-only DNA (Xu *et al.*, 2012 ▶). The *Xenopus laevis* Tet3 (xlTet3) CXXC domain shares a conserved sequence with the human TET3 CXXC domain and they exhibit similar DNA binding properties. Recently, two crystal structures of xlTet3-DNA complexes (PDB codes: 4hp3 and 4hp1) have been determined, which provided further insight into the mechanism of DNA binding (Xu *et al.*, 2012 ▶). In both xlTet3 CXXC domain complexes, the key residue His90 forms two hydrogen bonds, with the target cytosine (backbone carbonyl) and the complementary guanine (side chain ND1), respectively. In the structure of the xlTet3 CXXC domain with the CmCGG containing dsDNA, Ser89 of the CXXC domain flips back the peptide plane to accommodate the guanosine, which pairs with 5mC, and the Gln91 side chain becomes disordered to accommodate the complementary methylated cytosine. Therefore, this complex uncovered the mechanism of the xlTet3 CXXC domain binding to non-CpG DNAs, such as CpA and CpT. It was also found that the binding mode is conserved in the human TET3 CXXC domain by sequence alignment and ITC binding experiments (Xu *et al.*, 2012 ▶).

## 10. CD domain   

**Figure 4:**
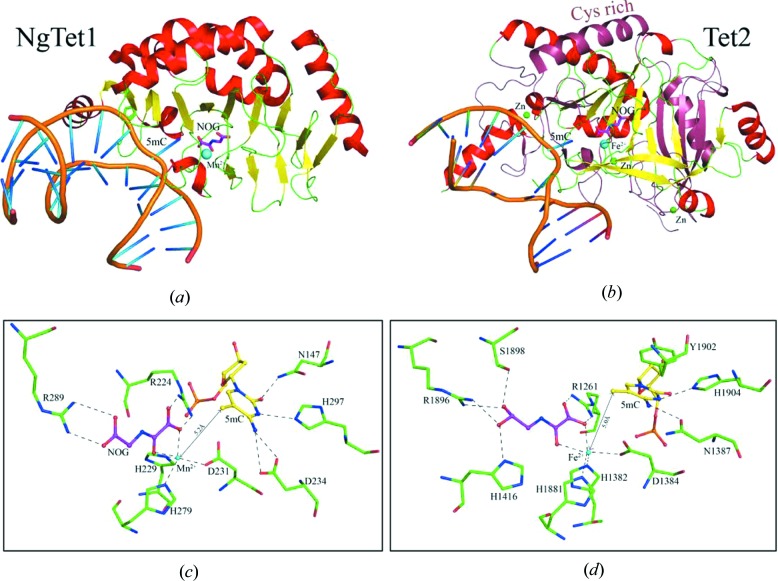
Structural characterization of TET family. Cartoon representation of (*a*) *Naegleria* TET1–DNA–NOG–Mn^2+^ complex (PDB code: 4lt5) and (*b*) human TET2–DNA–NOG–Fe^2+^ complex (PDB code: 4nm6), respectively. NOG is shown in sticks with carbon in magenta. Cofactor Mn^2+^ or Fe^2+^ is shown as cyan spheres. Three zinc atoms are shown as green spheres. Catalytic substrate 5mC is labeled. Close-up view of the coordination of the cosubstrate NOG, cofactor Mn^2+^/Fe^2+^ and catalytic substrate 5mC in the active sites of (*c*) NgTet1 and (*d*) human TET2. The hydrogen bonds are displayed as dashed lines and the distance between Mn^2+^/Fe^2+^ with 5mC is labeled.

The *Naegleria* Tet-like protein 1 (NgTet1), the homolog of mammalian TET1, can use 5mC, 5hmc or 5fC as substrates to generate 5cac. Recently, the complex crystal structures of NgTet1–DNA–NOG–Mn^2+^ (NOG, a 2-OG analog) and the TET2–DNA–NOG–Fe^2+^ have been determined (Hashimoto *et al.*, 2014 ▶; Hu *et al.*, 2013 ▶), and they adopt similar DSBH folding with the DNA located on the basic surface (Figs. 4 ▶ *a* and 4*b*). However, in the TET2 structure, the Cys-rich domain wraps around the DSBH domain to maintain the structural and catalytic integrity, and three zinc atoms further stabilize the overall structure (Fig. 4 ▶ *b*). Both of them use a base-flipping mechanism to target the methylated cytosine which results in a distortion of the B-form DNA duplex of ∼65° and ∼40° in NgTet1 and TET2, respectively. The flipped 5mC is stabilized *via* hydrogen bonds formed with three residues (Asn147, His297 and Asp234 in NgTet1, and Asn1387, His1904 and Tyr1902 in TET2), but the methyl group is not involved in any interaction (Figs. 4 ▶ *c* and 4*d*). In addition, the distance between the target methyl group and metal ion (∼5.2 Å in NgTet1 and ∼5.0 Å in TET2) is able to accommodate the hydroxyl (Figs. 4*c* and 4*d*), formyl or carboxylate group other than methyl, consistent with the TET family being active on 5mC-oxidized derivatives (5hmc, 5fmc or 5cac) as a substrate or product. Surprisingly, these 5mC-interacting residues are crucial for the catalytic activity of NgTet1 and TET2, but not important for DNA-binding affinity in TET2 (Hu *et al.*, 2013 ▶; Hashimoto *et al.*, 2014 ▶).

## 11. RNA demethylase   

RNAs serve vital roles in a wide variety of cellular processes (Sharp, 2009 ▶). There is increasing evidence that the modified nucleotides are involved in RNA metabolism. To date, more than 100 distinct types of post-transcriptional modifications have been characterized in tRNA, rRNA, mRNA and other RNAs (Cantara *et al.*, 2011 ▶; Machnicka *et al.*, 2013 ▶). Methyl­ation of the adenosine N6 position (m^6^A) is the most common internal mRNA modification in eukaryotic cells and viruses (Grosjean, 2005 ▶). The m^6^A modification is mostly found in the conserved motif Pu[G>A]m^6^AC[A/C/U], and the m^6^A sites have been identified in about 7000 mRNA transcripts and 300 noncoding RNA (Wei & Moss, 1977 ▶; Dominissini *et al.*, 2012 ▶). Furthermore, m^6^A sequencing studies showed that the m^6^A modifications preferentially occur near the stop codon, 3′UTR and the long internal exons (Meyer *et al.*, 2012 ▶; Dominissini *et al.*, 2012 ▶). The methyltransferase METTL3 and METTL14 (‘writer’) are employed to catalyze the mRNA methylation (Liu *et al.*, 2013 ▶). Knockdown of methyltransferase in embryonic stem (ES) cells leads to the loss of self-renewal capability, suggesting the importance of m^6^A modification in maintaining the properties of ES cells (Wang, Li *et al.*, 2014 ▶; Minton, 2014 ▶). The YTH domain containing proteins YTHDF1–3 are found to serve as ‘readers’ and bind the m^6^A. Recent studies showed that the ‘readers’ influence the re­localization of mRNA to affect the stability of the mRNA **in vivo** (Wang, Lu *et al.*, 2014 ▶).

The research on RNA methylation (m^6^A) has been stimulated by the characterization of RNA demethyl­ases (‘eraser’): fat mass and obesity-associated protein (FTO) and alkylation protein AlkB homolog 5 (ALKBH5), suggesting that the modifications are reversible and dynamic (Jia *et al.*, 2011 ▶; Zheng *et al.*, 2013 ▶). Both FTO and ALKBH5 are members of the AlkB subfamily of iron(II)/2-oxoglutarate-dependent dioxygenases.

## 12. FTO   

FTO is highly expressed in the brain and is mainly localized in the nucleus; a low level of FTO can also be detected in the cytoplasm (Meyer & Jaffrey, 2014 ▶). The FTO gene is associated with the body mass index (BMI) and type 2 diabetes (Wehr *et al.*, 2010 ▶; Frayling *et al.*, 2007 ▶; Dina *et al.*, 2007 ▶). Overexpression of FTO in mice leads to the increase of both food intake and body weight (Church *et al.*, 2010 ▶). Whereas the *Fto*-deficient mice display lean phenotypes even with an increased food intake (Fischer *et al.*, 2009 ▶; Church *et al.*, 2009 ▶). Notably, the conserved residue R316Q mutant (which recognizes the 2OG) causes postnatal growth retardation, thereby demonstrating that demethylase activity is associated with growth and development (Boissel *et al.*, 2009 ▶; Gulati & Yeo, 2013 ▶). Furthermore, more recent studies have demonstrated that FTO is also involved in glucose metabolism, Alzheimer’s disease, neurological diseases, cancer, *etc*. (Lappalainen *et al.*, 2011 ▶; Keller *et al.*, 2011 ▶; Kaklamani *et al.*, 2011 ▶; Tarabra *et al.*, 2012 ▶). However, the molecular mechanisms underlying the role of FTO in these physiological processes remain unclear. Besides m^6^A, FTO also demethyl­ases m^3^T of single-stranded DNA (ssDNA) and m^3^U of single-stranded RNA (ssRNA) (Jia *et al.*, 2008 ▶; Gerken *et al.*, 2007 ▶).

**Figure 5:**
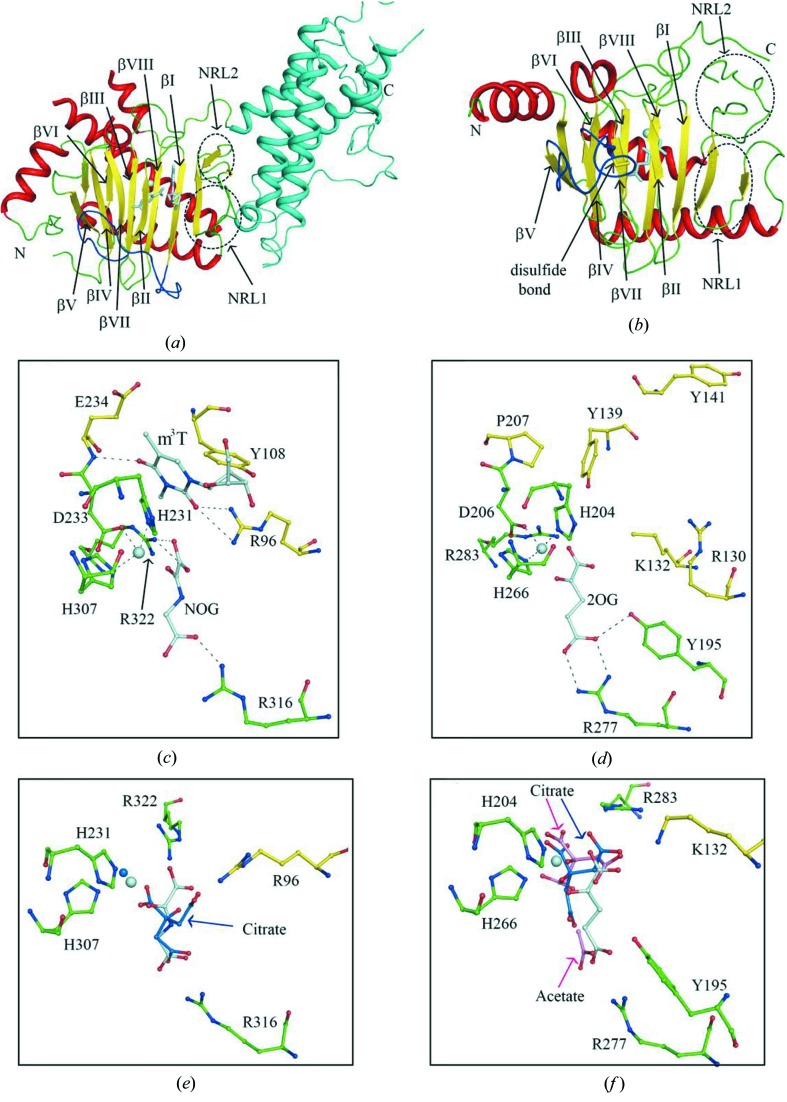
Structural characterization of RNA demethylases. (*a*) Cartoon representation of FTO in complex with m^3^T (PDB code: 3lfm). The β-strands in the DSBH fold are labeled. Key features highlighted are NRL1 (dotted circle), NRL2 (dotted circle), βI–βII loop (blue) and CTD (cyan). (*b*) Cartoon representation of ALKBH5 in complex with 2OG (PDB code: 4oct). The disulfide bond is shown in stick representation and labeled. (*c*) Close-up view of the ligand-binding sites in FTO. The conserved HxD/E…H…R…R motif and the ligands are shown in green and pale cyan, respectively. The metal ion is shown in sphere mode. The interactions are displayed as dashed lines. (*d*) Close-up view of the ligand-binding sites in ALKBH5. (*e*) Schematic of the FTO–citrate interaction (PDB code: 4ie7). The citrate is shown as stick and colored in blue. (*f*) Schematic of the ALKBH5–citrate interaction (PDB codes: 4o61 and 4nrm). The citrate molecules from two structures are colored blue and pink, respectively.

In 2010, Han and colleagues determined the crystal structure of FTO in complex with m^3^T (Han *et al.*, 2010 ▶). The overall structure can be divided into two domains, namely the N-terminal domain (NTD) and C-terminal domain (CTD) (Fig. 5 ▶ *a*). Characteristic of the NTD is the jelly-roll motif that mediates RNA demethylase activity. The canonical HxD/E…H…R…R motif in NTD (H231–D233–H307–R316–R322) is employed to coordinate the metal ion and NOG. The nucleobase of the substrate is stacked on the conserved residues Tyr108 and His231 (Fig. 5 ▶ *c*). The carbonyl oxygen of substrate is further stabilized through the hydrogen-bond interactions with polar residues (Arg96 and Glu234), defining the substrate specificity. The most prominent feature in FTO is the βI–βII loop that links the minor β-sheet and major β-sheet. The βI–βII loop (Fig. 5 ▶ *a*), the unique insertion in FTO members compared with other AlkB proteins, covers the substrate binding pocket and enables FTO to discriminate between dsDNA and ssDNA (or ssRNA).

The CTD of FTO folds into a helix-bundle structure, and makes extensive contact with NTD, especially the nucleotide recognition lid (NRL). *In vitro*, neither the NTD or CTD domain alone showed the demethylase activity. The results were consistent with the FTO mutations that disrupt the NTD–CTD interactions. However, the activity was rescued by mixing CTD into NTD fractions, suggesting the key roles of CTD in FTO activity. It is likely that CTD contributes to the stability and orientation of the lid region, and thereby activates the demethylation activity.

### 12.1. ALKBH5   

ALKBH5 is another RNA demethylase identified **in vivo** and **in vitro** (Zheng *et al.*, 2013 ▶). ALKBH5, encoded on the chromosome 17p11, is predominantly localized in the nucleus and is highly expressed in testis (Both *et al.*, 2012 ▶; Zheng *et al.*, 2013 ▶). The hypoxia inducible factor-1 (HIF-1) and protein arginine methyltransferase 7 (PRMT7) have been shown to play a role in regulating the ALKBH5 gene expression (Feng *et al.*, 2013 ▶; Thalhammer *et al.*, 2011 ▶). Recent studies suggested that ALKBH5 influences nuclear mRNA export and RNA synthesis. In *Alkbh5*-deficient cells, the level of m^6^A in mRNA is increased. Although the *Alkbh5*-null mice are viable, the mouse lacking ALKBH5 displays a sterile phenotype, indicating that ALKBH5 is essential for fertility (Zheng *et al.*, 2013 ▶).

Unlike FTO, ALKBH5 only catalyzes the demethylation of m^6^A, but not other RNA modifications in ssRNA or ssDNA. Most recently, four groups have independently solved the crystal structures of ALKBH5 (Xu *et al.*, 2014 ▶; Feng *et al.*, 2014 ▶; Chen *et al.*, 2014 ▶; Aik *et al.*, 2014 ▶). The overall structure of ALKBH5 displays a DSBH fold (Fig. 5 ▶ *b*). The catalytic triad HxD/E…H in ALKBH5 (H204–D206–H266) coordinates the metal ion. The conserved residues arginine (Arg277) and aromatic residue (Tyr195) are hydrogen bonded to 2OG (Fig. 5 ▶ *d*). Unlike other Alkb members, another conserved arginine (Arg283) appears not to form the direct interaction with 2OG. Structural alignment and modeling showed that the nucleobase might be bracketed between Tyr139 (or Tyr141) and His204, and basic residues (Arg130 and/or Lys132) further contribute to stabilize the nucleobase *via* hydrogen-bond interactions.

The βIV–βV loop has the function of discrimination against dsDNA. Additionally, the βIV–βV loop is anchored to the minor β-sheet *via* the conserved disulfide bond in ALKBH5 proteins, determining the conformation of the βIV–βV loop (Fig. 5 ▶ *b*). In the absence of a disulfide bond, ALKBH5 showed the demethylation activity towards dsDNA, suggesting the crucial role of the disulfide bond in ssDNA (or ssRNA) preference (Feng *et al.*, 2014 ▶). However, without a disulfide bond, the βIV–βV loop in zebrafish ALKBH5 undergoes a conformational change and would also clash with the dsDNA (Chen *et al.*, 2014 ▶). Further experiments are needed to investigate the role of the disulfide bond.

## 13. Structural and functional comparison   

Not surprisingly, both FTO and ALKBH5 share the DSBH fold and the conserved HxD/E…H…R…R motif. The intermediates are present in the demethylation process of FTO other than ALKBH5 (Fu *et al.*, 2013 ▶). The substrates in FTO and ALKBH5, stacking between the aromatic and histidine rings, are involved in interactions with basic residues. Furthermore, an acidic residue from the βII–βIII loop (Glu235 in FTO) also contributes to the interaction with the nucleobase. The equivalent position to Glu235 in other Alkb proteins is a polar residue, with some exceptions, where it is a neutral residue (Pro207) in ALKBH5 (Figs. 5*c* and 5*d*). Therefore, it is tempting to speculate that the nucleobase recognition modes between ALKBH5 and FTO are distinct. These interactions with nucleobase could endow substrate preference on AlkB proteins. Interestingly, swapping the βII–βIII loop between the AlkB demethylases can lead to an exchange of demethylation activities (Zhu & Yi, 2014 ▶), indicating the key roles of the βII–βIII loop in substrate specificity.

The NRLs occur at the similar position in both FTO and ALKBH5. Despite the close similarities with the NRL2 (*e.g.* high flexibility), the NRL1 in both proteins is strikingly different. A β-strand and a long loop that is close to the substrate-binding pocket constitute the NRL1 in FTO. However, the NRL1 in ALKBH5 consists of two β-strands, exposing an open space over the substrate-binding pocket (Figs. 5*a* and 5*b*). The differences, taken together, are responsible for substrate preference.

Although they both employ a loop to discriminate against dsDNA, the positions of the loops are distinct. Moreover, the corresponding loop in FTO is an insertion in comparison with other AlkB members. The C-terminal region is important for FTO demethylaton activity; however, the ALKBH5 variant carrying the C-terminal region deletion had no significant effect on demethylaton activity. Of note, the phosphorylation of the C-terminal region may be involved in the ALKBH5 function or stability.

FTO and ALKBH5 displayed distinct inhibition profiles against the generic 2OG dioxygenase inhibitors. IOX3 is a more potent inhibitor of FTO than 2,4-PDCA. By contrast, 2,4-PDCA is the most potent ALKNH5 inhibitor (Aik *et al.*, 2013 ▶; Chen *et al.*, 2012 ▶; Xu *et al.*, 2014 ▶; Feng *et al.*, 2014 ▶; Aik *et al.*, 2014 ▶). In the presence of a citrate buffer, the 2OG-binding pockets of both the ALKBH5 and FTO are occupied by the citrate molecule, which is a modest inhibitor of ALKBH5 and FTO. In FTO, the citrate molecule occurs at the analogous position of 2OG; however, in ALKBH5, the citrate molecule is found at a different position to that in 2OG. Moreover, the metal ion is absent in ALKBH5–citrate complex structure, but the metal ion is still present in FTO–citrate complex structure (Figs. 5*e* and 5*f*). These distinct binding modes may be helpful in developing selective inhibitors of AlkB proteins.

## 14. Concluding remarks   

Dioxygenases consist of many subgroups, including Fe^II^ and 2OG-dependent dioxygenases and FAD-dependent amine oxidases. In eukaryotes, these two classes of dioxygenases play an important role in regulating gene expression by catalyzing the demethylation of histones, DNAs or RNAs. Much progress has been made to advance our knowledge about the biological functions of these dioxygenases and their implications in human diseases, which would contribute to better and faster identifying and validating these targets for therapeutics. Without any doubt, there is still much more to be investigated about this superfamily and its involvement in epigenetics in the future.
